# Low-Loading f-MXene/Fluorosilicone Hybrid Highly Hydrophobic Coatings: Anti-Photoaging Mechanism and Application in Durable Protection of Stone and Brick Cultural Heritage

**DOI:** 10.3390/polym18111346

**Published:** 2026-05-29

**Authors:** Peng Fu, Shaojun Yan, Kaili He, Meirong Shi

**Affiliations:** Shaanxi Institute for the Preservation of Cultural Heritage, Xi’an 710075, China; 15609296307@163.com (S.Y.); hekaili1209@163.com (K.H.); shimeirong123@163.com (M.S.)

**Keywords:** Ti_3_C_2_T_X_ MXene, fluorosilicone coating, stone cultural heritage, anti-photoaging, high hydrophobicity

## Abstract

In the surface protection of stone and brick cultural heritage, a primary challenge is that traditional polymeric coatings are prone to photooxidative degradation under ultraviolet (UV) irradiation, and the resulting aged fragments readily block the substrate micropores, leading to a loss of “breathability”. To address the performance conflict among waterproofing, breathability, and weather resistance, this study prepared few-layer Ti_3_C_2_T_X_ MXene using a minimally intensive layer delamination (MILD) method. The poor compatibility between MXene and the fluorosilicone (FPS) resin matrix was effectively resolved through covalent modification with a silane coupling agent (KH-550). Results demonstrate that at an ultralow loading (0.5 wt%), the functionalized f-MXene is uniformly dispersed within the resin. This structure not only spontaneously constructs a hierarchical rough architecture on the surface that imparts high hydrophobicity (water contact angle of 131.6°), but its internal “labyrinth effect” also effectively blocks corrosive media. Simultaneously, the intrinsic water vapor transmission rate of the substrate is effectively maintained (with a reduction of less than 3%), and no visually perceptible color difference is generated (∆E = 1.2). Mechanically, f-MXene relies on interfacial interactions to act as a “nano-skeleton” for stress transfer, thereby increasing the uniaxial compressive strength of fragile limestone by 32.4%. Optical and spectroscopic characterizations further elucidate its anti-aging mechanism: f-MXene not only provides broadband UV shielding but also exhibits highly efficient radical scavenging activity during long-term UV aging. After 400 h of aging, the concentrations of hydroxyl and superoxide anion radicals within the system are significantly reduced, blocking the photooxidative chain reaction from the source. This work develops a composite protective material system for stone cultural heritage that simultaneously integrates high moisture permeability, minimal visual intervention, and long-term antioxidant performance.

## 1. Introduction

Immovable stone and brick cultural heritage (e.g., ancient pagodas, grotto temples, and historical city walls) serves as vital material archives chronicling the evolution of human civilization [1,2]. However, prolonged exposure to extreme open-air environments renders these highly porous inorganic structures highly susceptible to severe environmental degradation, including capillary water infiltration, soluble salt crystallization weathering, freeze-thaw cycles, and biodegradation [3,4,5]. Among these deterioration driving factors, liquid water acts not only as a medium but also as the core catalyst for the vast majority of destructive physicochemical reactions [6,7]. Therefore, constructing hydrophobic protective coatings on the surface of cultural relics to impede the transfacial mass transfer of external moisture and corrosive ions at the source remains the most direct and effective paradigm for maintaining the structural stability of ancient architecture and implementing preventive conservation [8,9]. The rigorous evaluation of such protective treatments for porous inorganic cultural heritage relies heavily on standardized testing protocols and guidelines established by the European Committee for Standardization [10,11,12,13,14,15].

Currently, commercial organic polymeric materials (such as polysiloxanes, fluorocarbon resins, and acrylates) are widely applied in the surface consolidation and waterproofing of stone cultural heritage due to their superior initial hydrophobicity and film-forming processability [16,17], and green enzymatically synthesized biobased latexes with excellent thermal stability provide a sustainable alternative for such applications [18]. However, under long-term practical service environments, these traditional polymer coatings exhibit severe intrinsic limitations [19,20]. Under the coupled excitation of continuous ultraviolet (UV) irradiation, thermal stress, and environmental oxygen, the polymer matrix inevitably undergoes photooxidative degradation and macromolecular chain cleavage, which macroscopically manifests as severe yellowing, embrittlement, and microcrack initiation in the coatings [21,22]. More critically, the degraded polymer fragments readily block the stone and brick micropores, sharply reducing the water vapor transmission rate (WVTR) of the material [23,24]. This sealing effect accelerates the stress accumulation of liquid water and crystallized salts at the coating–substrate interface, ultimately triggering catastrophic chalking and large-scale exfoliation of the heritage surface [25]. Consequently, resolving the inherent contradiction between the “long-term weather resistance” and “substrate compatibility” (i.e., high vapor permeability and minimal visual intervention) of protective coatings represents a major challenge in contemporary cultural heritage conservation science [26,27].

To retard the degradation kinetics of organic protective materials, incorporating nanofillers (such as SiO_2_, ZnO, and TiO_2_) to construct organic–inorganic composite systems has emerged as a mainstream strategy in recent years [28,29]. However, traditional semiconductor nanoparticles possess high photocatalytic activity under UV excitation, rendering them prone to inducing abundant reactive oxygen species (ROS) radicals, which paradoxically accelerates the autocatalytic degradation of the surrounding polymer matrix. On the other hand, although carbon-based two-dimensional materials like graphene exhibit excellent physical barrier properties, their strong visible light absorption characteristics irreversibly alter the intrinsic color of cultural heritage, severely violating the principles of “minimum intervention” and “visual authenticity” established in the international heritage conservation community [30,31]. Therefore, there is an urgent need to develop novel nanofillers that concurrently provide broadband UV shielding, high chemical inertness, and colorless transparency to light irradiation at an ultralow loading.

Two-dimensional transition metal carbides and nitrides (MXenes), as an emerging class of 2D layered materials, offer an unprecedented opportunity to break through the aforementioned bottlenecks [32,33,34]. Among them, the most extensively studied Ti_3_C_2_T_X_ features an extremely high geometric aspect ratio and abundant surface terminal groups (e.g., -OH, -O, -F) [35,36,37]. Its unique 2D lamellar skeleton can construct a highly complex “tortuous path” effect within the polymer matrix, effectively retarding the permeation of water molecules and corrosive media in physical space [38,39]. More crucially, distinct from traditional fillers, Ti_3_C_2_T_X_ not only possesses excellent narrow-bandgap UV absorption capabilities, but its surface reductive functional groups also exhibit remarkable radical scavenging activity. This enables the termination of the polymer’s photooxidative chain reaction from the source without triggering any secondary photocatalytic damage [40,41]. Although MXene has shown great promise in heavy-duty anti-corrosion and electromagnetic shielding domains [42,43], its introduction into the field of cultural heritage conservation—which imposes exceptionally stringent requirements on environmental tolerance and aesthetic compatibility—remains a highly promising and underexplored scientific frontier.

Driven by the aforementioned challenges in stone and brick cultural heritage conservation, this study proposes a novel multi-functional synergistic protection strategy to meet the demand for multi-performance coordinated optimization of heritage protective coatings. On the basis of existing studies focusing on the single performance optimization of MXene-polymer waterproof/corrosion-resistant coatings [44,45] and traditional anti-UV nanocomposite systems [46,47], this strategy realizes the synergistic enhancement of multiple core protective functions at an ultralow nanofiller loading, achieving the balanced optimization of waterproofing, breathability, UV resistance, and long-term weather resistance of the protective coating. Utilizing an improved minimally intensive layer delamination (MILD) technique and silanization interfacial engineering, this study covalently anchors functionalized Ti_3_C_2_T_X_ MXene (f-MXene)—which fully retains its broadband UV absorption properties and surface reductive sites—into a hydrophobic resin cross-linking network at an ultralow loading, thereby constructing a high-strength organic–inorganic hybrid topological skeleton. In terms of interfacial mass transfer barrier, the directionally aligned 2D nanosheets within the matrix significantly prolong the diffusion path of liquid water and corrosive media, achieving highly efficient water blockage without significantly affecting the intrinsic water vapor transmission rate (WVTR) of the porous substrate, while effectively dissipating the interfacial thermal stress induced by drastic fluctuations in environmental temperature and humidity. Regarding anti-photooxidative degradation, f-MXene leverages its outstanding radical scavenging activity to continuously terminate the polymer photooxidative chain reaction induced by high-energy UV radiation from the source. This work breaks the performance trade-off limits among high-efficiency water resistance, porous substrate compatibility (high moisture permeability and minimal color difference intervention), and long-term weather resistance of protective materials, providing a universal theoretical basis for the durable preservation and preventive conservation of stone and brick cultural heritage under complex environmental stresses.

## 2. Experimental Section

### 2.1. Reagents and Chemicals

The Ti_3_AlC_2_ MAX phase precursor powder (purity > 99%) was purchased from Jilin Yiyi Technology Co., Ltd. (Changchun, China). Analytical grade reagents, including lithium fluoride (LiF, 99%), concentrated hydrochloric acid (HCl, 36–38%), 3-aminopropyltriethoxysilane (KH-550, 99%), absolute ethanol, xylene, and isopropanol, were all acquired from Sinopharm Chemical Reagent Co., Ltd. (Shanghai, China). Commercial fluorosilicone (FPS) resin was obtained from Yun Fu Technology Co., Ltd., Xi’an, China. Deionized (DI) water was used throughout the experiments.

Black bricks and limestone, representing typical ancient architecture and immovable stone cultural heritage, were selected as the testing substrates. After being cut into standard dimensions (e.g., 50 mm × 50 mm × 10 mm), the substrates were sequentially ultrasonically cleaned with DI water and absolute ethanol, and then dried to a constant weight in an oven at 60 °C for subsequent use.

### 2.2. Preparation and Functionalization of f-Ti_3_C_2_T_X_ MXene

Preparation of Ti_3_C_2_T_X_ MXene via the MILD method: First, 1.6 g of LiF was slowly added to 20 mL of concentrated HCl (9 M) in a polytetrafluoroethylene (PTFE) beaker and magnetically stirred for 30 min to form a homogeneous etching solution. Subsequently, under an ice-bath condition, 1.0 g of Ti_3_AlC_2_ MAX phase powder was gradually added to the aforementioned mixture in batches. The reaction system was then transferred to a 45 °C water bath and continuously stirred for 24 h to selectively etch the Al atomic layers. Upon completion of the reaction, the acidic suspension in the centrifuge tubes was repeatedly washed with DI water and centrifuged (3500 rpm, 5 min per cycle) until the pH of the supernatant approached 6. Afterwards, the precipitate was resuspended in DI water, subjected to ultrasonic delamination in an ice bath for 1 h, and centrifuged at 3500 rpm for 10 min. The dark green supernatant, rich in few-layer Ti_3_C_2_T_X_ MXene, was collected and lyophilized to obtain the pristine MXene powder.

Surface functionalization of f-MXene with KH-550: In total, 200 mg of the prepared pristine MXene powder was ultrasonically dispersed in 40 mL of a mixed solvent of ethanol and DI water (3:1 by volume). Then, 0.5 mL of the KH-550 silane coupling agent was slowly added dropwise using a microsyringe. The mixture was refluxed and vigorously stirred under an argon atmosphere at 60 °C for 12 h. After the reaction, the product was collected via centrifugation, washed three times with absolute ethanol to remove unreacted free silane, and finally dried in a vacuum oven at 60 °C for 12 h to yield the hydrophobically modified f-MXene nanosheets.

### 2.3. Preparation of Coatings and Specimens

At a designated mass fraction of 0.5 wt%, the f-MXene powder was added to a xylene/isopropanol mixed solvent and ultrasonically dispersed for 30 min to form a uniform suspension. Subsequently, the FPS resin base was slowly added to the suspension, followed by magnetic stirring for 2 h to ensure the thorough dispersion of the nanosheets within the polymer matrix. Then, tetraethoxysilane (TEOS, as crosslinker) and dibutyltin dilaurate (DBTDL, as catalyst) were added. The dosage of TEOS was 5 wt% of the FPS resin, and DBTDL was 0.5 wt% of the total resin mass. The mixture was continuously stirred until homogeneous. For the consolidation and protection of the black brick and limestone substrates, the aforementioned composite coating was uniformly applied to the substrate surfaces via brushing (or spraying), with the coating amount controlled at approximately 150 g/m^2^. The treated specimens were naturally cured at room temperature (25 °C, 50% relative humidity) for 7 days. Subsequent performance tests were conducted once the system was fully cross-linked and cured. The pure FPS coating was prepared using the same procedure to serve as control groups. Free-standing coating films were obtained by casting the mixture into PTFE molds, curing, and subsequently peeling them off.

### 2.4. Mechanical Property Testing

Tensile strength refers to the maximum tensile force that a specimen of a specific width can withstand during the stretching process, serving to evaluate the film’s ability to resist external tensile failure. The free-standing coating films were cut into standard dimensions and subjected to tensile testing using a universal testing machine at a preset extension rate. The tensile strength (N) was recorded when the film specimen fractured during the test. For the consolidated limestone specimens, uniaxial compressive strength (MPa) tests were performed using the universal testing machine.

### 2.5. UV Aging Test

The treated coating specimens were placed in a UV fluorescent aging test chamber equipped with UVA-340 lamps to conduct accelerated aging tests under condensation cycles. The sample surfaces were maintained at a fixed distance from the UV lamp plane and were continuously exposed in the chamber for 400 h (for color difference and contact angle measurements) and 1000 h (for free radical concentration analysis) to evaluate their long-term weather resistance.

### 2.6. Water Vapor Transmission Rate and Hydrophobicity Testing

Water Vapor Transmission Rate Testing: The WVTR was determined using the gravimetric method in accordance with relevant standards. Parallel samples were tested for 24 h under constant temperature and humidity conditions (23 °C, 50% relative humidity). The experiment was characterized by mass loss (g), and the WVTR was calculated in g·m^−2^·24 h^−1^. A higher mass loss indicates greater water vapor permeability of the sample.

Hydrophobicity Testing: The static water contact angle (WCA) of the coating surfaces was measured using a contact angle goniometer. DI water droplets were dispensed onto the sample surfaces, and the contact angle magnitude was recorded and quantified to assess the liquid water repellency of the coatings before and after modification.

Water Absorption Capillarity Test: Specimens (50 mm × 50 mm × 10 mm) were dried in an oven at 60 °C to constant mass (mass change ≤0.1% over 24 h), then cooled to (23 ± 1) °C in a desiccator before testing. A saturated bedding layer (filter paper, thickness ≥ 5 mm) was placed in a water container. The dried specimen was weighed (m_0_) and placed with its testing surface downward on the saturated layer. Water was added to keep the level constant without submerging the specimen. The mass of the specimen was measured at fixed time intervals (10, 30, 60, 120, 180, 240 min). After each weighing, surface water was gently wiped off with a damp cloth [10].

Qi = (m_i_ − m_0_)/A

where m_0_ is the mass of the specimen at time t_0_, in kilograms; mi is the mass of the specimen at time ti, in kilograms; A is the area of the specimen in contact with the bedding layer (cotton), in meters squared.

### 2.7. Color Difference Testing

A portable spectrophotometer was utilized to conduct color difference tests on the specimens. The CIE L*a*b* color coordinate system was employed to characterize the color variations on the substrate surfaces. A smaller color difference value (∆E*) indicates more imperceptible color changes in the samples before and after coating and accelerated aging. The color difference in the CIE L*a*b* color system was calculated using the following formula:

∆E* = [(∆L*)^2^ + (∆a*)^2^ + (∆b*)^2^]^1/2^

where ∆L* represents the difference in lightness, ∆a* represents the red-green color deviation, and ∆b* represents the yellow-blue color deviation. Initially, the L*, a*, and b* values of a standard sample were measured, and then the color difference conclusions were drawn by comparing the discrepancies between the sample values and the standard values.

### 2.8. Instruments

The crystalline structure, microscopic morphology, and surface composition of the samples were physicochemically characterized using a scanning electron microscope (SEM, SU8010, Hitachi High-Technologies Corporation, Tokyo, Japan) equipped with an energy-dispersive spectrometer (EDS), an X-ray diffractometer (XRD, D8 Advance, Bruker AXS GmbH, Karlsruhe, Germany), a Fourier transform infrared spectrometer (FTIR, Nicolet iS50, Thermo Fisher Scientific, Waltham, MA, USA), and an X-ray photoelectron spectrometer (XPS, ESCALAB 250Xi, Thermo Fisher Scientific, Waltham, MA, USA). The optical absorption and physical shielding properties of the films were measured using a UV–visible (UV–Vis, UV-2600, Shimadzu Corporation, Kyoto, Japan) spectrophotometer, while the internal reactive species after aging were detected utilizing an electron paramagnetic resonance (EPR, A300, Bruker BioSpin GmbH, Rheinstetten, Germany) spectrometer.

## 3. Results and Discussion

### 3.1. Structural Characterization of f-MXene

**Scheme 1:**
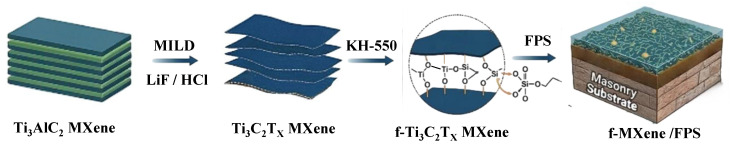
Schematic of the fabrication process of functionalized Ti_3_C_2_T_X_ MXene and the corresponding composite protective coating.

In this study, high-quality few-layer Ti_3_C_2_T_X_ MXene nanosheets were successfully synthesized from the Ti_3_AlC_2_ MAX phase precursor using an improved, minimally intensive layer delamination method with LiF/HCl as the etching system. This mild etching strategy selectively removes the Al atomic layers from the MAX phase lattice while effectively suppressing the excessive oxidation of MXene nanosheets during the etching and ultrasonic delamination processes, thereby maximizing the preservation of the intrinsic structure and physicochemical properties of the two-dimensional layered material. To address the hydrophilicity caused by the abundant hydroxyl (-OH) and fluorine (-F) terminal groups on the MXene surface and its poor compatibility with non-polar fluorocarbon/siloxane matrices, a silane coupling agent (KH-550) was introduced for surface functionalization in this study (Scheme 1).

**Figure 1:**
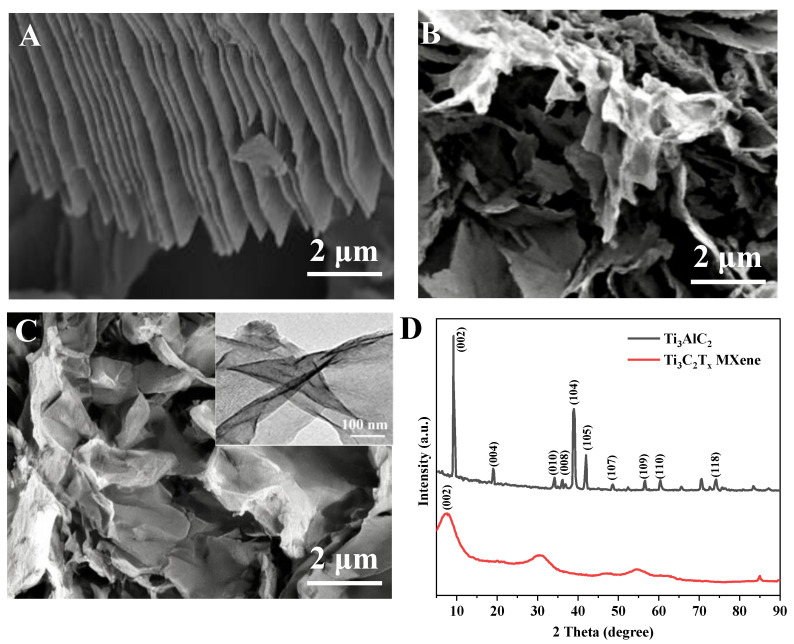
(**A**) SEM image of Ti_3_AlC_2_ MAX phase. (**B**) SEM image of Ti_3_C_2_T_X_ MXene. (**C**) SEM image of f-MXene. (**D**) XRD spectra of Ti_3_AlC_2_ and Ti_3_C_2_T_X_ MXene.

SEM was employed to systematically characterize the morphological evolution of the materials before and after etching and delamination, with the results shown in Figure 1. The unetched Ti_3_AlC_2_ MAX phase precursor exhibits a typical accordion-like, tightly stacked layered bulk structure (Figure 1A). Following selective etching and ultrasonic delamination in an ice bath, the bulk MAX phase is completely exfoliated into independent two-dimensional Ti_3_C_2_T_X_ MXene nanosheets (Figure 1B). The MXene powder modified by the silane coupling agent (f-MXene) presents an ultrathin, cicada-wing-like morphology characteristic of 2D layered materials, with a clean and flat surface (Figure 1C). Neither obvious residual polymer phase particles nor TiO_2_ nanoparticles generated by excessive oxidation were observed, verifying the high quality and structural integrity of the prepared f-MXene nanosheets.

XRD was utilized to quantitatively verify the crystal structure evolution of the materials before and after etching and delamination, and the results are presented in Figure 1D. After etching and modification, the strong characteristic diffraction peak of the f-MXene phase located at 9.5° corresponding to the (104) crystal plane completely disappears, indicating that the Al atomic layers in the MAX phase skeleton have been completely removed. Meanwhile, the characteristic diffraction peak of the (002) crystal plane, corresponding to the layered stacking structure, shifts significantly to lower angles from 9.5° in the precursor to 6.2°. Calculated according to Bragg’s law 2*d sin θ* = *nλ* (where the wavelength of the Cu Kα radiation *λ* = 0.15406 nm, *n* is the diffraction order, and *θ* is the Bragg diffraction angle), the interlayer spacing of the material expands significantly from 0.93 nm in the pristine Ti_3_AlC_2_ to 1.42 nm in the exfoliated f-MXene. This substantial increase in interlayer spacing not only further corroborates that the bulk MAX phase was successfully etched and exfoliated into few-layer MXene nanosheets but also confirms that a large number of solvent molecules and oxygen-containing terminal groups (e.g., -OH, -O) have successfully intercalated between the MXene layers. These abundant surface active sites provide ample reaction sites for the silanization covalent grafting and interfacial modification of MXene.

To overcome the intrinsic bottlenecks of pristine Ti_3_C_2_T_X_ MXene—namely, its strong surface hydrophilicity, poor compatibility with non-polar resin matrices, and severe tendency to induce abundant reactive oxygen species (ROS) radicals—KH-550 was employed for covalent grafting. The terminal ethoxy groups of KH-550 hydrolyze into highly reactive silanols (Si-OH) in a weakly acidic system, which subsequently undergo dehydration condensation with the abundant Ti-OH terminals on the MXene surface. This establishes stable Ti-O-Si covalent bonds, anchoring the organic silane chains onto the 2D lamellae. Although KH-550 selectively reacts with -OH groups rather than the highly polar -F terminals, the covalently grafted long alkyl chains form a dense organic network that exerts a strong steric shielding effect, physically preventing water molecules from interacting with the underlying -F groups and thereby ensuring the macroscopic organophilicity of f-MXene.

**Figure 2:**
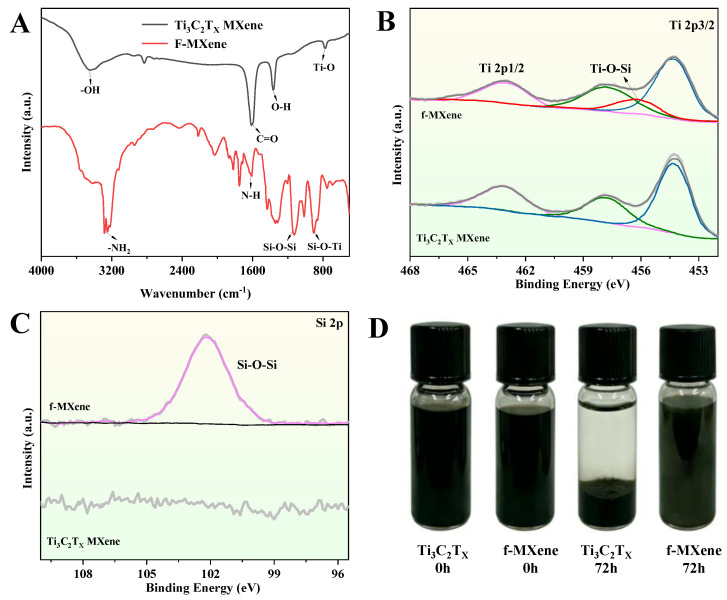
(**A**) FTIR spectra, (**B**) XPS Ti 2p spectrum, (**C**) XPS Si 2p spectrum of Ti_3_C_2_T_X_ MXene and f-MXene. (**D**) Digital photograph of the suspension stability.

FTIR spectroscopy directly corroborates this covalent grafting (Figure 2A). Pristine MXene exhibits broad O-H stretching (3200–3600 cm^−1^) and adsorbed water bending (~1630 cm^−1^) vibrations, alongside characteristic Ti-C (~580 cm^−1^) and Ti-O (~550 cm^−1^) skeletal peaks. Following modification, the f-MXene spectrum displays a substantial attenuation of the -OH and water peaks, confirming the extensive consumption of surface hydroxyls and the consequent reduction in hydrophilicity [48]. Crucially, the emergence of the Si-O-Ti stretching peak at ~920 cm^−1^ and the intensified Si-O-Si peak at ~1100 cm^−1^ provide definitive evidence of covalent bonding, clearly distinguishing it from a physical admixture. The intact Ti-C peak verifies the preservation of the intrinsic 2D skeleton. Furthermore, the appearance of -NH_2_ (3300 and 1570 cm^−1^) and alkyl C-H (2930 and 2860 cm^−1^) vibrations validates the successful anchoring of KH-550 chains, with the reactive terminal amino groups well preserved [49].

To quantitatively elucidate the chemical basis of the interfacial coupling at the atomic level, XPS analysis was conducted. Within the Ti 2p spectrum (Figure 2B), a novel characteristic peak assigned to the Ti-O-Si linkage emerges at 468.2 eV following the modification. Coupled with the evidence from the Si 2p signal (Figure 2C) and the signal originating from the amino terminus of KH-550, this conclusively substantiates that the organic molecular chains have been successfully covalently grafted onto the surface of the 2D nanosheets. Consequently, the modified f-MXene undergoes a fundamental transition from hydrophilicity to pronounced organophilicity. It maintains a stable suspension in common organic solvents (isopropanol and xylene) for over 72 h without stratification, laying a crucial material foundation for the subsequent construction of homogeneous, transparent composite protective coatings for heritage conservation (Figure 2D).

### 3.2. Performance of f-Mxene/FPS Composite Coating

Hydrophobicity is of paramount importance for the conservation of open-air ancient brick and stone architecture as well as movable stone relics, given that liquid water acts as the primary transport vehicle and prerequisite trigger for myriad irreversible deteriorations, including substrate dissolution, salt crystallization weathering, acidic media erosion, deleterious ion migration, and microbial biodeterioration. Consequently, the liquid water repellency of a coating constitutes the core determinant of its surface protective efficacy and long-term service durability.

**Figure 3:**
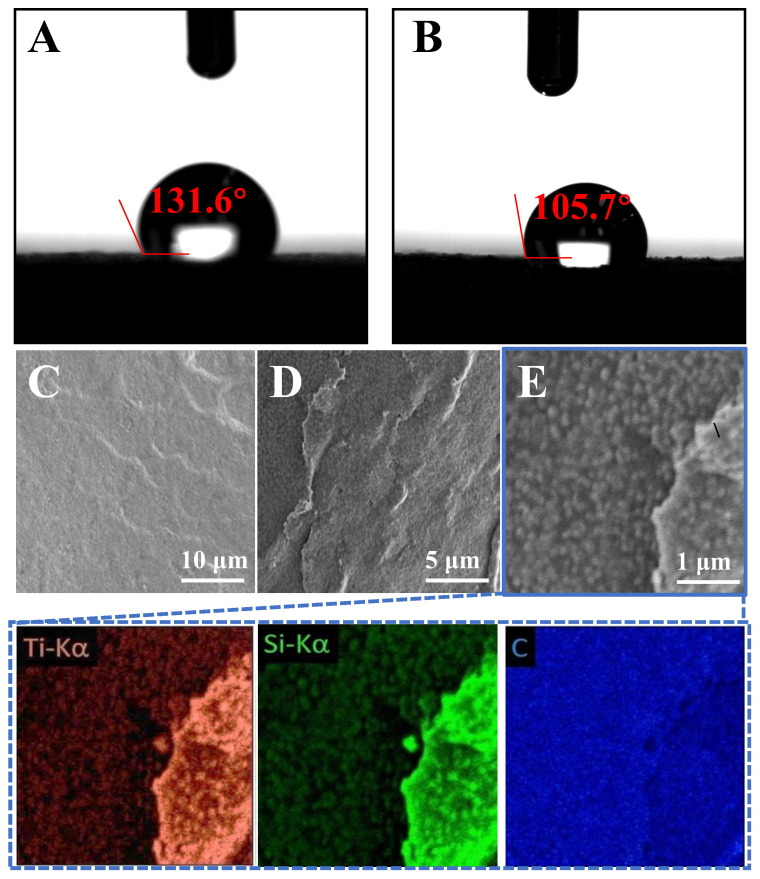
(**A**) WCA of the f-Mxene/FPS composite coating. (**B**) WCA of the FPS coating. (**C**,**D**) SEM images of the f-Mxene/FPS composite coating. (**E**) EDS elemental mapping of the f-Mxene/FPS composite coating.

Even at an ultralow f-MXene loading of 0.5 wt% within the resin matrix, the resultant f-MXene/resin composite protective coating exhibits significantly enhanced hydrophobicity on the black brick substrate. As evidenced by the static WCA measurements (Figure 3A), deionized water droplets form a nearly ideal spherical shape on the composite-coated brick substrate, achieving a WCA of 131.6°. This represents a substantial elevation compared to the 105.7° exhibited by the pure resin control coating (Figure 3B). This fundamental improvement in hydrophobicity is directly corroborated by microstructural and elemental distribution characterizations. SEM observations reveal that the silane-functionalized f-MXene nanosheets are uniformly dispersed within the resin matrix without discernible agglomeration. During the film-forming and curing process, these homogeneously distributed 2D nanosheets spontaneously construct a step-like micro/nano-hierarchical rough structure on the substrate surface (Figure 3C,D). Energy-dispersive X-ray spectroscopy (EDS) elemental mapping further substantiates that the Ti element (originating from the MXene skeleton), Si element (from the grafted silane chains), and C element (from the resin matrix) exhibit a uniform co-distribution across the tested area without localized aggregation (Figure 3E). This directly verifies the excellent dispersion compatibility of f-MXene within the resin matrix at the elemental level, providing fundamental support for the uniform construction of the surface micro/nano-rough architecture.

Based on the Cassie–Baxter wetting model, the multiscale micro/nano-hierarchical structure on the coating surface can stably entrap a substantial volume of air at the solid–liquid interface, forming a continuous air-cushion layer [50,51]. This effectively minimizes the actual solid–liquid contact area between the water droplet and the coating surface, ultimately endowing the composite coating with high hydrophobicity, low adhesion, and self-cleaning properties, thereby establishing a highly efficient waterproof barrier for stone and brick heritage substrates. To further quantitatively verify the uniform dispersion of f-MXene and the micro-nano hierarchical structure of the coating, we performed atomic force microscopy (AFM) characterization, with detailed 3D topography and roughness parameters (Ra = 48.2 nm, Rq = 59.7 nm) provided in the Figure S1.

**Figure 4:**
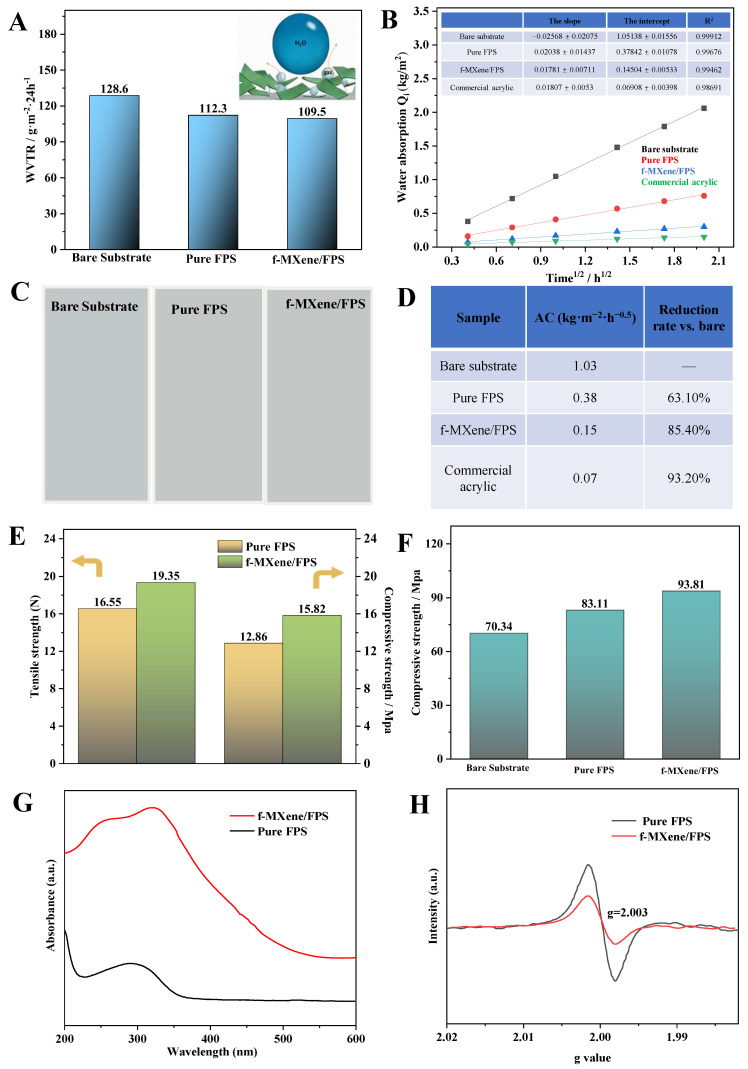
(**A**) WVTR, The inset shows the breathability mechanism of the f-MXene/FPS composite coating: the arrows indicate that water vapor and gas molecules can permeate and escape through the porous structure of the coating, while liquid water is effectively blocked. (**B**) Capillary water absorption kinetics. (**C**) AC values and (**D**) color difference of the bare substrate, pure FPS, and f-MXene/FPS. (**E**) Mechanical properties of the pure FPS and f-MXene/FPS. (**F**) Compressive strength of limestone consolidated by pure FPS and the composite coating. (**G**) UV–Vis absorption spectra and (**H**) EPR spectra of pure FPS and f-MXene/FPS.

The critical performance boundary of protective coatings for cultural heritage conservation lies in the retention of the WVTR, which is pivotal to avoiding irreversible secondary deteriorations, such as salt efflorescence and cracking, induced by internal moisture accumulation within the substrate. Utilizing the standard gravimetric method, parallel samples were tested for 24 h at 23 °C and 50% relative humidity. The WVTR experiment is characterized by mass loss (g); a higher mass loss indicates greater water vapor permeability of the sample. As depicted in Figure 4A, the WVTR of the bare black brick substrate is 128.6 g·m^−2^·24 h^−1^. Following treatment with the pure FPS coating, the WVTR is 112.3 g·m^−2^·24 h^−1^, representing a 12.5% decrease compared to the bare substrate. The WVTR of the 0.5 wt% f-MXene composite coating is 109.5 g·m^−2^·24 h^−1^, exhibiting only a 14.8% decrease relative to the bare substrate, with a marginal reduction of less than 3% compared to the pure resin coating, indicating no statistical difference. In stark contrast, treatment with a commercial traditional acrylic protective coating yields a substrate WVTR of merely 42 g·m^−2^·24 h^−1^, a severe 67.2% decline from the bare substrate, forming a highly impermeable interface. The aforementioned results substantiate that the uniformly dispersed f-MXene exclusively constructs the hierarchical rough structure requisite for high hydrophobicity on the coating surface, without obstructing the intrinsic water vapor diffusion channels of the fluorosilicone cross-linked network. Consequently, it effectively maintains the moisture exchange capacity between the stone and brick substrate and the external environment while significantly impeding liquid water infiltration.

The capillary water absorption kinetics further validate the superior protective efficacy of the f-MXene/FPS coating. As shown in Figure 4B,D, the bare black brick exhibited a high capillary absorption coefficient (AC) of 1.03 kg·m^−2^·h^−0.5^. After treatment with the f-MXene/FPS composite, the AC value plummeted to 0.15 kg·m^−2^·h^−0.5^, representing an 85.4% reduction compared to the bare substrate. This confirms that the hierarchical rough architecture and high surface energy barrier effectively suppress the capillary suction of liquid water. Notably, although the commercial acrylic coating showed the lowest AC (0.07 kg·m^−2^·h^−0.5^), its extreme pore-sealing effect resulted in a catastrophic loss of breathability (67.2% reduction in WVTR). In contrast, the f-MXene/FPS system achieves an optimal balance, providing robust liquid water resistance while maintaining excellent substrate compatibility.

Furthermore, benefiting from the ultralow loading (0.5 wt%) of f-MXene and its homogeneous dispersion within the fluorosilicone matrix, the composite coating exerts no significant adverse effects on the intrinsic properties of the resin (Figure 4C). Color difference test results demonstrate that the ∆E of the black brick substrate post-treatment is 1.2 ± 0.3, which is below the threshold of human visual perception (∆E < 3) and exhibits no statistical difference from the pure fluorosilicone coating (∆E = 1.0). This thoroughly satisfies the requirement of minimal visual intervention for cultural heritage conservation.

Mechanical characterization of the free-standing films reveals that the introduction of f-MXene substantially enhances the intrinsic mechanical properties of the FPS resin. Its tensile strength increases from 16.54 N for the pure resin to 19.35 N, and the compressive strength of the film steadily grows from 12.86 MPa to 15.82 MPa (Figure 4E). This reinforcement effect is primarily attributed to the uniform dispersion of the high-aspect-ratio f-MXene nanosheets within the matrix. Tightly bonded with the polymer chains via chemical bonds established by the KH-550 coupling agent, they act as a “nano-skeleton” to effectively bear and dissipate external stress, thereby significantly optimizing the fracture and deformation resistance of the protective film.

In the consolidation tests conducted on limestone substrates, the composite coating demonstrates a significant reinforcement effect, elevating the average uniaxial compressive strength of the samples from 71.15 MPa of the pristine substrate to 94.2 MPa (Figure 4F). This dramatic increase of 32.4% far exceeds the enhancement afforded by the pure resin coating. This outcome proves that the f-MXene/FPS system not only provides dense surface protection for stone cultural heritage but also achieves deep structural consolidation of fragile minerals by optimizing the stress transfer at the coating–stone interface. This holds substantial application value for enhancing the structural stability of ancient buildings and stone relics under self-weight, weathering, and seismic loads.

To deeply elucidate the enhancement mechanism of f-MXene on the long-term weather resistance of the FPS coating, UV–Vis absorption spectroscopy and EPR techniques were employed to systematically characterize the anti-photooxidative aging performance of the composites from the dual perspectives of physical shielding and chemical quenching. As illustrated in the UV–Vis absorption spectra in Figure 4G, the pure FPS resin exhibits extremely low absorption intensity in the 200–400 nm UV region, rendering it nearly transparent. This implies that high-energy UV photons can easily penetrate the pure resin protective film, reaching the interior of the material and triggering the photodegradation of polymer chains. However, upon the incorporation of f-MXene nanosheets, the absorbance of the f-MXene/FPS composite coating exhibits a dramatic leap across the entire UV spectrum (especially in the high-energy UVC/UVB region of 200–350 nm), displaying broad-band absorption characteristics. This phenomenon indicates that f-MXene, with its unique band structure and lamellar morphology, constructs a dense “UV shielding network” within the polymer matrix. Through intense physical absorption and multiple interlayer scattering, it effectively obstructs the penetration of UV photons into the deep substrate and the protected heritage, endowing the composite coating with an outstanding primary physical protective barrier.

Beyond passive physical shielding, the long-term stability of the coating relies more heavily on the active scavenging of generated destructive free radicals. Figure 4H presents the EPR spectra of the materials during the aging process. The signal at g = 2.003 is typically assigned to the spin adducts of reactive oxygen species (ROS), such as hydroxyl radicals (·OH) and superoxide anion radicals (·O_2_^-^), generated during the photooxidative aging of the polymer [52,53]. The test results reveal that the pure FPS system exhibits extremely high free radical characteristic peak intensity, indicating that it is undergoing a severe photooxidative chain cleavage reaction internally. In sharp contrast, the resonance peak intensity of the f-MXene/FPS composite coating is drastically attenuated. This remarkable signal suppression (i.e., a substantial reduction in radical concentration) directly confirms that f-MXene plays a critical role as an efficient “radical scavenger” within the system. f-MXene can rapidly and continuously quench primary free radicals induced by trace amounts of penetrating UV light, thereby powerfully terminating the oxidative degradation cycle of the polymer chains from the source. Semi-quantitative analysis reveals that the incorporation of 0.5 wt% f-MXene reduces the concentration of trapped reactive oxygen species (ROS) by approximately 60% compared to the pure FPS matrix.

### 3.3. Characterization of Properties for Cultural Heritage Conservation Applications

When evaluating the practical application value of protective materials, their weather resistance during long-term service is of paramount importance. In this study, the long-term chemical stability of the composite coatings was quantitatively evaluated through a 400 h artificial accelerated ultraviolet (UV) aging experiment (UV-condensation cycle).

**Figure 5:**
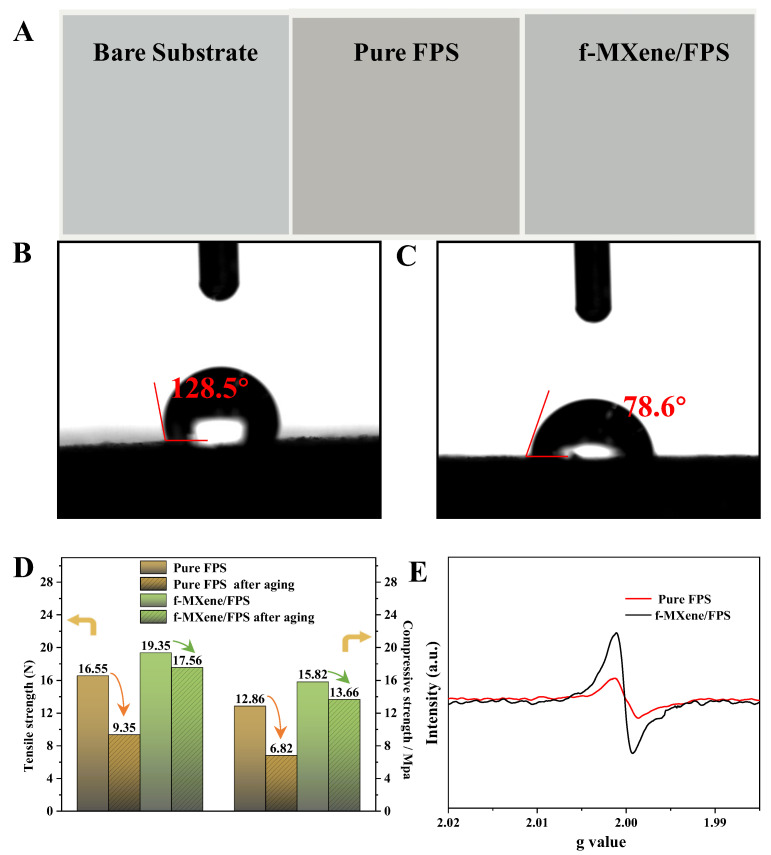
(**A**) Color difference of the pure FPS coating and f-MXene/FPS composite coating after aging. (**B**) WCA of the pure FPS coating and (**C**) f-MXene/FPS composite coating after aging. (**D**) Mechanical properties and (**E**) EPR test of the pure FPS film and f-MXene/FPS composite film after aging.

As shown in the colorimetric stability test results in Figure 5A, ∆E of the pure resin coating exceeded the human visual perception threshold (∆E = 3) and ultimately reached up to 8.5 after 400 h of accelerated aging. This phenomenon stems from the photooxidative chain reaction of polymer molecular chains under the action of high-energy UV photons, leading to chain cleavage and the massive generation of chromophoric groups containing carbonyl and carboxyl functionalities, which ultimately results in irreversible yellowing of the coating. In stark contrast, the 0.5 wt% f-MXene composite coating exhibited excellent colorimetric stability throughout the entire aging cycle. After 400 h of aging, its ∆E was merely 2.2, consistently remaining below the human visual perception threshold and demonstrating no statistical difference from the color difference level of the pure resin coating before aging.

Regarding the long-term efficacy of wettability and protective functions, the static WCA test results of the coatings before and after aging are presented in Figure 5B. After 400 h of aging, the static WCA of the pure resin coating drastically attenuated from an initial 105.7 to 78.6°, indicating a substantial loss of its hydrophobic protective capability. Conversely, the static WCA of the 0.5 wt% f-MXene composite coating remained at 128.5° post-aging, representing a hydrophobicity retention rate exceeding 96% and preserving its high hydrophobicity, low-adhesion, and self-cleaning characteristics. This significant performance retention originates from two synergistic mechanisms: First, the abundant reductive functional groups on the f-MXene surface continuously exert a radical scavenging effect, terminating the polymer’s photooxidative chain reaction and preventing the extensive generation of polar hydrophilic groups. Second, the “nano-reinforcement” effect of the 2D nanosheets suppresses the initiation and propagation of microcracks during the coating aging process, thereby maintaining the micro/nano-hierarchical rough structure required for high hydrophobicity and ensuring the stability of the waterproof protective function during long-term service.

As illustrated in Figure 5D, the comparison of mechanical properties after accelerated aging further underscores the critical contribution of f-MXene to the long-term weather resistance of the coating. Following severe UV aging tests, the mechanical properties of the pure FPS film experienced a precipitous decline, with its tensile strength and compressive strength plummeting to 9.35 N and 6.82 MPa, respectively. Macroscopically, this manifested as severe embrittlement induced by photooxidative chain cleavage. In contrast, benefiting from the potent anti-aging mechanism of the 2D nanosheets, the f-MXene/FPS composite film exhibited superior structural stability. Its tensile strength (17.56 N) and compressive strength (13.66 MPa) after aging remained at remarkably high levels, demonstrating an excellent mechanical property retention rate. This significant data comparison directly corroborates that f-MXene can effectively obstruct the degradation pathway of the polymer matrix under long-term environmental stress, thereby ensuring the structural integrity and mechanical load-bearing capacity of the cultural heritage protective materials during prolonged service.

Furthermore, EPR test results further validated the radical scavenging mechanism during long-term aging (Figure 5E): after aging, the characteristic peak intensities of the spin adducts for ·OH and·O_2_^-^ in the pure resin coating system remained at a high level, whereas the radical concentration in the f-MXene composite coating system was significantly lower. This result confirms that during long-term UV irradiation, f-MXene consistently maintains highly efficient radical scavenging activity, continuously terminating the photooxidative chain reaction of the polymer, and successfully achieving the core decoupling of “long-term weather resistance” and “substrate compatibility” for protective coatings.

## 4. Conclusions

In this study, through molecular-level interfacial modification technology, an ultralow loading of f-MXene was homogeneously dispersed into FPS resin to successfully develop a nanocomposite coating dedicated to the durable protection of porous stone and brick cultural heritage. Investigations reveal that the uniform dispersion and parallel orientation of f-MXene within the matrix not only construct a hierarchical rough structure on the surface that imparts high hydrophobicity (water contact angle of 131.6°). The composite coating exhibits a 14.8% reduction in WVTR relative to the bare substrate, but importantly, it induces a negligible additional WVTR reduction (<3%) compared to the pure resin coating, effectively maintaining the substrate’s intrinsic breathability.

Moreover, it induces no visually perceptible color alteration (∆E = 1.2), successfully achieving an optimal balance between highly efficient waterproofing and substrate breathability. Regarding structural consolidation, f-MXene leverages interfacial Si-O-Ti covalent bonds to function as a “nano-skeleton” for efficient stress transfer, propelling the average uniaxial compressive strength of the consolidated fragile limestone specimens from 71.15 MPa to 94.2 MPa—a substantial surge of 32.4% that demonstrates remarkable mechanical reinforcement. More critically, optical and spectroscopic characterizations validate a dual anti-photooxidative aging mechanism governed by UV shielding and radical quenching. This mechanism fundamentally terminates the degradation chain reaction at the source, enabling the aged coating to retain 96% of its initial hydrophobicity and 87.5% of its hardness, alongside a highly stable colorimetric performance (∆E = 2.2). In summary, the proposed f-MXene/FPS composite coating strictly adheres to the “minimum intervention” principle of cultural heritage conservation. It effectively surmounts the inherent flaws of traditional protective materials, specifically their tendency to seal substrate micropores and their poor weatherability, thereby providing a highly translatable advanced material paradigm for the long-term preservation of immovable porous inorganic cultural heritage.

Future research will prioritize long-term in situ field monitoring and multi-factor coupled aging assessments to further evaluate the material’s full life-cycle stability and reversibility in complex real-world environments. This will ensure the sustainable and safe application of f-MXene/FPS coatings in the preventive conservation of immovable cultural heritage.

## Data Availability

The original contributions presented in this study are included in the article/Supplementary Material. Further inquiries can be directed to the corresponding author.

## Supplementary Materials

The following supporting information can be downloaded at: https://www.mdpi.com/article/10.3390/polym18111346/s1, Figure S1: 3D AFM topography of the f-MXene/FPS composite coating (scanning area: 5 μm × 5 μm). The arithmetic mean roughness (Ra) of the coating is 48.2 nm, and the root mean square roughness (Rq) is 59.7 nm; Table S1: Performance of f-MXene/FPS composite coatings with different f-MXene loadings.
