# Supplementary material for: Low-Loading f-MXene/Fluorosilicone Hybrid Highly Hydrophobic Coatings: Anti-Photoaging Mechanism and Application in Durable Protection of Stone and Brick Cultural Heritage
Source: Polymers (Basel). 2026 May 29;18(11):1346. doi: 10.3390/polym18111346 (PMC13258994; doi:10.3390/polym18111346)
Supplement: Supplementary file 1 [file polymers-18-01346-s001.zip › polymers-4278357-supplementary.pdf]

Supporting Information

Low-Loading f-MXene/Fluorosilicone Hybrid Highly Hydrophobic Coatings: Anti-Photoaging Mechanism and Application in Durable rotection of Stone and Brick Cultural Heritage

Peng Fu*, Shaojun Yan., Kaili He, Meirong Shi.

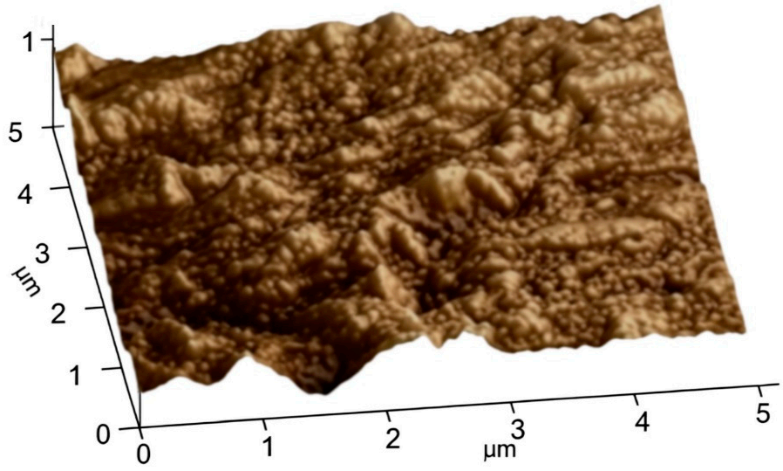

Figure S1. 3D AFM topography of the f-MXene/FPS composite coating (scanning area: 5 µm × 5 µm). The arithmetic mean roughness (Ra) of the coating is 48.2 nm, and the root mean square roughness (Rq) is 59.7 nm.

Table S1. Performance of f-MXene/FPS composite coatings with different f-MXene loadings

f-MXene Loading (wt%)	Water Contact Angle (WCA, °)	Color Difference (ΔE)	Dispersion State (Visual)
**0.1**	112.4 ± 1.5	0.8 ± 0.2	Uniform
**0.3**	123.5 ± 1.2	1.6 ± 0.3	Uniform
**0.5 (Optimal)**	131.6 ± 1.8	2.6 ± 0.4	Uniform, no obvious agglomeration
**1.0**	125.2 ± 2.4	5.8 ± 0.6	Slight agglomeration
**2.0**	115.8 ± 3.1	11.4 ± 1.2	Severe agglomeration / Restacking

For cultural heritage applications, maintaining the original visual appearance is paramount (typically requiring a color difference ΔE < 3). Due to the inherent dark coloration of MXene, loadings exceeding 0.5 wt% (e.g., 1.0% and 2.0%) resulted in unacceptable darkening of the substrate (ΔE > 5). The 0.5 wt% loading (ΔE = 2.6) safely meets the conservation standards.

From a materials science perspective, when the concentration exceeds 0.5 wt%, the strong van der Waals forces between 2D nanosheets induce severe restacking and agglomeration within the FPS matrix. This disrupts the uniform micro/nano-hierarchical structure, causing the hydrophobicity to actually decrease from its peak of 131.6° down to 115.8° at 2.0 wt%.

Therefore, 0.5 wt% represents the optimal threshold to maximize protective performance while strictly adhering to visual preservation criteria. We have incorporated this quantitative justification into Section 2.3 and the Supplementary Materials of the revised manuscript. We thank you for prompting us to clarify this critical detail.
